# Renal ewing sarcoma in a young female: a case report and review of targeted therapy

**DOI:** 10.3389/fsurg.2025.1512474

**Published:** 2025-03-04

**Authors:** Pengfei Wang, Mingfa Wang, Jiangtao Zhan, Xinming Hu, Xusong Meng

**Affiliations:** ^1^ Hainan Medical University, Haikou, China; ^2^ Department of Pathology, The Second Affiliated Hospital of Hainan Medical University, Haikou, China; ^3^ Department of Urology, The Second Affiliated Hospital of Hainan Medical University, Haikou, China

**Keywords:** ewing sarcoma, renal neoplasms, P53, diagnosis, targeted therapy

## Abstract

Ewing sarcoma (ES) is an aggressive neoplasm predominantly affecting pediatric and adolescent populations. Renal involvement in ES is exceedingly rare, representing less than 1% of all renal malignancies. Herein, we present the case of a 22-year-old female diagnosed with renal Ewing sarcoma (RES) accompanied by renal vein thrombosis. The patient reported a one-month history of persistent left lumbar pain, prompting hospitalization. Magnetic resonance imaging identified an extensive left suprarenal mass measuring 13.5 × 10.5 × 4.5 cm, with concurrent renal vein thrombosis. The comprehensive evaluation of histopathology, immunohistochemistry and molecular genetics confirmed RES. The treatment included radical left nephrectomy, followed by adjuvant chemotherapy (i.e., vincristine, epirubicin and cyclophosphamide) after surgery. Genetic analysis of the tumor revealed mutations in P53 and STGA2. Follow-up contrast-enhanced computed tomography scans of the patient demonstrated metastatic progression to the pancreas. The patient passed away after a 7-month follow-up period. This article reviews our treatment experience and recent developments in targeted therapies. Aiming to provide new approaches for the treatment of RES, this combines next-generation sequencing technology with targeted therapy to promote the optimization of targeted treatments.

## 1 Introduction

Ewing sarcoma (ES) is a rare and aggressive malignancy characterized by a group of undifferentiated tumors derived from the neuroectoderm (1). Predominantly affecting the bones and soft tissues in pediatric and adolescent populations, primary kidney ES (RES) is exceedingly rare, accounting for less than 1% of all renal tumors (2). More than 65% of RES patients present with distant metastases at diagnosis, commonly involving the regional lymph nodes, lungs, and liver (3). Histopathologically, classical descriptions under light microscopy reveal small, round blue cells arranged in sheets or rosettes (4). Immunohistochemical analysis typically demonstrates positivity for CD99 and FLI-1 markers (1). The hallmark genetic alteration in ES, including renal involvement, is the characteristic chromosomal translocation t(11;22)(q24;q12) between the EWS gene on chromosome 22 and the FLI-1 gene on chromosome 11 (5). Current treatment guidelines recommend surgical resection followed by adjuvant chemotherapy. The standard chemotherapy regimen comprises a combination of vincristine, doxorubicin, and dactinomycin, supplemented by ifosfamide and etoposide (6). This report presents a rare case of RES and reviews the existing literature to elucidate the potential mechanisms and therapeutic prospects of targeted therapy in the management of these tumors.

## 2 Case presentation

### 2.1 Examination

**Figure 1:**
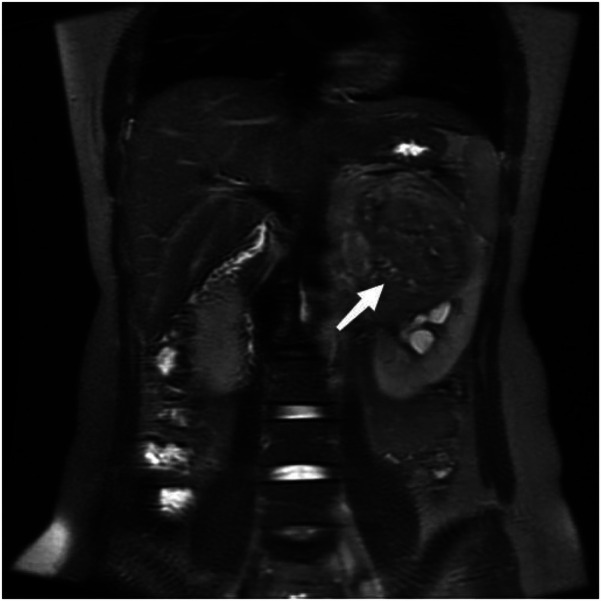
Coronal reconstruction of the tumor on a T2-weighted image, demonstrating tumor thrombus invasion into the left renal vein.

A 22-year-old female presented with a one-month history of recurrent left lumbar pain. Physical examination revealed left abdominal tenderness and a palpable mass. An abdominal computed tomography (CT) scan performed at an external facility identified a large tumor in the left kidney, initially suspected to be renal carcinoma; however, no therapeutic interventions were initiated at that time. The patient had no history of renal disease or cancer, and there was no family history of renal carcinoma. Upon admission, renal magnetic resonance imaging (MRI) was conducted, revealing a substantial tumor in the upper segment of the left kidney, measuring approximately 13.5 × 10.5 × 4.5 cm, associated with intratumoral hemorrhage and a tumor thrombus extending into the left renal vein (Figure 1).

Single-photon emission computed tomography (SPECT) imaging of the kidneys demonstrated a significant reduction in glomerular filtration rate (GFR) and effective renal plasma flow (ERPF) in the left kidney, with values of 7.9 ml/min and 11.1 ml/min, respectively. In contrast, the GFR and ERPF of the right kidney were within normal limits, recorded at 65.7 ml/min and 289.2 ml/min, respectively.

The tumor marker cancer antigen 125 (CA125) was elevated at 131 U/ml, while other tumor markers, including alpha-fetoprotein (AFP), carcinoembryonic antigen (CEA), and CA199, remained within normal ranges. Additionally, chest CT imaging revealed no other abnormalities.

**Figure 2:**
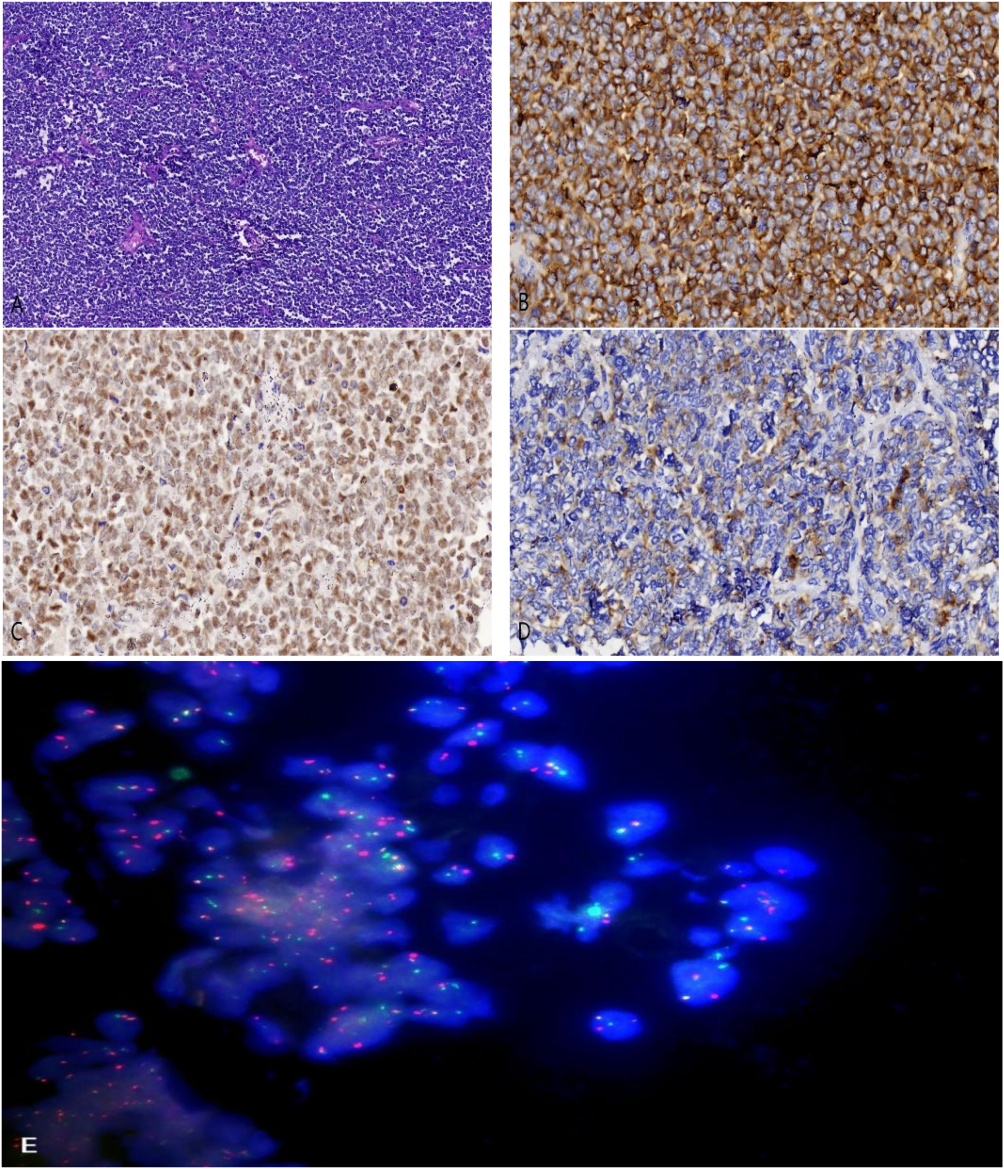
**(A)** hematoxylin and eosin staining revealing small malignant round cells arranged in sheets and nests (magnification, ×100). **(B)** Tumor cells showing positive staining for CD99 (magnification, ×400). **(C)** Tumor cells showing positive staining for FLI-1 (magnification, ×400). **(D)** Tumor cells showing positive staining for Synaptophysin (Syn) (magnification, ×400). **(E)** EWSR1 gene translocation was detected by fluorescence *in situ* hybridization testing (FISH).

Preoperative renal tumor biopsy was performed on the patient, with tumor tissue collected for pathological and fluorescence *in situ* hybridization (FISH) tests. Hematoxylin and eosin staining under light microscopy showed uniform small round cells with clear nuclei, prominent nuclear division, and cells arranged in sheets and nests with a focal rosette-like pattern (Figure 2A). Pathological examination confirmed the diagnosis of RES. Immunohistochemical staining revealed positivity for CD99, FLI-1, and Syn (Figures 2B–D), while staining for vimentin, CK, CgA, WT-1, and PAX-8 was negative. The detection of t(22q12): EWS-FLI1 type 1 translocation rearrangement through fluorescence *in situ* hybridization (FISH) further clarified the diagnosis (Figure 2E).

### 2.2 Surgical procedure

**Figure 3:**
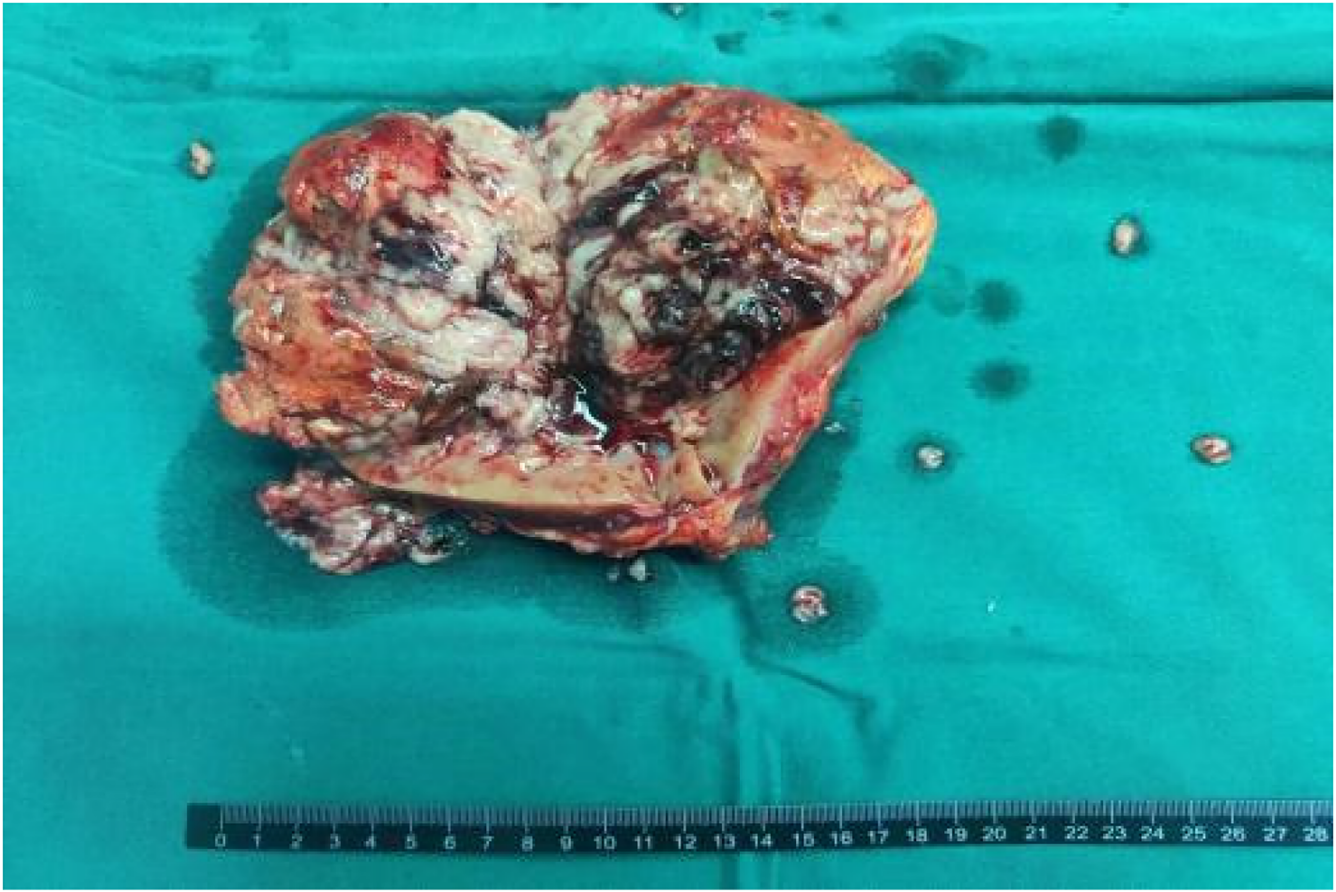
Gross appearance of the tumor, showing grey-white and grey-red areas with evident necrosis.

Subsequently, the patient underwent a radical nephrectomy. The tumor was measured to be 15 × 12.5 × 4 cm. Macroscopic examination revealed grey-white and grey-red masses, predominantly necrotic (Figure 3). Pathological analysis identified a tumor thrombus within the vein and evidence of vascular and nerve invasion in the left kidney. No tumor invasion was detected in the renal pelvis, capsule, adrenal gland, vessel ends, or ureter, and no metastasis was observed in the sampled lymph nodes (0/3).

### 2.3 Postoperative treatment and follow-up

**Figure 4:**
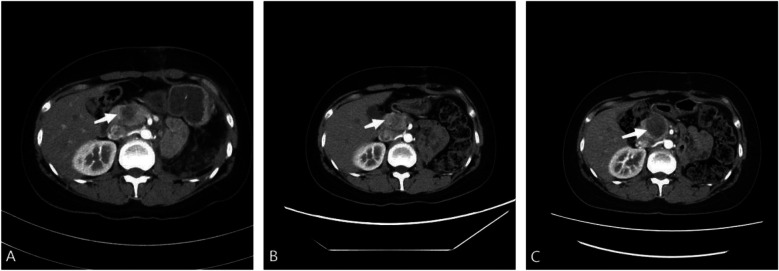
**(A)** post-operative enhanced computed tomography (CT) scan showing enlargement of the pancreatic head to approximately 30 × 15 mm, indicating a metastatic tumor. **(B)** After one cycle of adjuvant chemotherapy, enhanced CT scan showing reduction of the pancreatic head to approximately 20 × 12 mm. **(C)** After three cycles of adjuvant chemotherapy, enhanced CT scan showing enlargement of the pancreatic head to approximately 35 × 34 mm.

To develop a personalized treatment strategy, tumor tissues were embedded in paraffin and sent for genetic analysis, which detected mutations in the TP53 and STAG2 genes, with mutant allele frequencies of 31.62% and 3.17%, respectively. Based on these genetic findings, the laboratory recommended a regimen including gemcitabine, docetaxel, tegafur, and cyclophosphamide. Whole-body bone SPECT showed no significant abnormalities. Following the guidelines of the National Comprehensive Cancer Network (NCCN) and the Chinese Society of Clinical Oncology (CSCO), the patient underwent three cycles of adjuvant chemotherapy, administered every three weeks. The regimen consisted of vincristine at 2 mg/m^2^, epirubicin at 150 mg/m^2^, and cyclophosphamide at 1,400 mg/m^2^ on the first day of each cycle, followed by ifosfamide at 2 g/m^2^ and etoposide at 120 mg/m^2^ for five consecutive days. During chemotherapy, the patient developed agranulocytosis, necessitating the administration of a granulocyte colony-stimulating factor to mitigate grade 4 bone marrow suppression. Furthermore, the patient also developed renal anemia, which was cured symptomatically with erythropoietin. Subsequent enhanced CT scans during chemotherapy showed fluctuating sizes of pancreatic metastases (Figure 4). Unfortunately, the patient did not receive further treatment after completing three cycles of chemotherapy and passed away 7 months after surgery.

## 3 Discussion

ES is the second most common primary bone malignancy in children and adolescents (7). While ES primarily arises in bones and soft tissues, it has also been documented in extraskeletal locations such as the kidney, ureter, urinary bladder, uterus, and cervix (8). However, occurrences of this tumor within the urogenital tract are exceptionally rare. RES was first described by Seemayer et al. in 1975, with fewer than 200 cases reported globally to date (9). The average age of patients with RES is 30.4 years, with approximately 60% being male (10). RES are particularly aggressive compared to other neuroectodermal tumors, often presenting as locally advanced or metastatic disease at diagnosis. Therefore, patients typically have a poor prognosis. According to a study by Rowe et al., 44% of patients with RES had metastases at initial presentation (11). Studies have shown that the average survival time for patients with advanced metastatic disease is 26.14 months, with a lower median survival time (12).

The clinical manifestations and imaging characteristics of RES are nonspecific, often leading to diagnostic challenges. For example, in our case, the initial diagnosis of renal cell carcinoma (RCC) was based solely on radiological findings. Common clinical presentations of ES include lower back pain, hematuria, and an abdominal mass. Radiologically, RES is typically characterized by a large, low-enhancement mass with multiple septa-like structures and peripheral hemorrhage (13). RCC is the most prevalent renal malignancy, predominantly affecting older adults. In contrast, enhanced CT imaging of RES typically shows lower enhancement during the nephrographic and cortical phases, more indistinct margins, greater internal necrosis, and a higher propensity for invasion into the renal veins and inferior vena cava (9).

The definitive diagnosis of RES was mainly based on histopathological, immunohistochemical and molecular genetic analyses. Histologically, the tumor cells are primitive, small, round, and blue, arranged in sheets and nests, with a high nuclear-cytoplasmic ratio and scant cytoplasm. Homer-Wright rosette formations are observed in several cases (14). Immunohistochemically, diffuse membranous expression of CD99 and FLI-1 is highly specific for this malignancy (15). CD99, a cell surface glycoprotein encoded by the MIC2 gene (16), is expressed in more than 90% of ES cases (17). Approximately 90% of ES or primitive neuroectodermal tumors (PNETs) exhibit a specific t(11;22)(q24;q12) translocation, resulting in the fusion of the EWS and FLI-1 genes and overexpression of the FLI-1 protein. Folpe et al. reported that FLI-1 specificity in ES exceeds 90% (18).

Given the rarity of RES, no standardized therapeutic regimen has been established. However, a literature review suggests that a multidisciplinary approach, involving surgical resection followed by adjuvant chemotherapy, is recommended. This typically involves a radical nephrectomy followed by postoperative chemotherapy (15). When tumor disorders impact young girls, it is critical to maintain the long-term function of their reproductive system and apply fertility-preserving treatment techniques (19). Chemotherapy is considered an essential adjuvant treatment for patients with ES. Common chemotherapeutic regimens include agents such as vincristine, ifosfamide, doxorubicin, etoposide, cyclophosphamide, and actinomycin D (1). ES is relatively sensitive to chemotherapy, and patients with ES can derive significant benefits from this treatment (20). In recent years, neoadjuvant chemotherapy following biopsy has been approved as a therapeutic strategy for ES. Typically, patients undergo preoperative chemotherapy for approximately 8–12 weeks to achieve local control, followed by surgical resection with the goal of achieving negative margins. Postoperative chemotherapy is then administered, with the possible addition of radiation therapy, particularly in cases with metastatic involvement (21). However, the efficacy of this treatment approach needs further validation.

Despite the recognized importance of chemotherapy in managing ES, the effectiveness of conventional regimens remains limited. Even with aggressive treatment strategies, the median overall survival for patients with primary RES is approximately 26.5 months (22). About 25% of patients with ES develop metastatic disease, with a survival rate of less than 30% (23). The efficacy of chemotherapy regimens is often hampered by the development of multidrug resistance, which is a major factor contributing to treatment failure in many patients (24). Therefore, there is an urgent need for novel therapeutic strategies aimed at improving patient survival while minimizing the adverse effects associated with traditional chemotherapy approaches.

Targeted therapy represents a groundbreaking approach in the management of cancer (25). This strategy has been shown to significantly enhance prognosis, reduce treatment-related toxicities, and offer viable alternatives for patients who cannot undergo conventional chemotherapy (26). With ongoing research, an increasing number of antitumor agents are being developed for advanced cases. In the presented case, genetic analysis identified mutations in the TP53 and STAG2 genes, with allele frequencies of 31.62% and 3.17%, respectively. A study by Liu et al. involving 99 ES patients indicated that high mutational burdens in TP53 and STAG2 correlate with poorer overall survival and shorter time to progression (27). According to a study by Tirode et al., patients with Ewing's sarcoma who have STAG2 mutations have considerably lower survival rates than those with TP53 mutations. Patients with both genetic alterations in their malignancies had the worst prognosis, whereas those without STAG2 and TP53 mutations had the highest survival rates (28). Given the extensive role of P53 in various cancers, it has become a prime target for new anticancer drugs.

P53, often termed the “guardian of the genome,” is a pivotal tumor suppressor gene involved in DNA repair, regulation of the cell cycle at the G1 checkpoint, apoptosis, and cellular senescence (29). Mutations in P53 are common across a range of cancers, found in approximately half of all human tumors (23). Evidence suggests that most P53 mutations result in the loss of its tumor suppressor functions, with some mutations contributing to tumorigenesis and progression (30). Although P53 mutations are found in about 10% of ES cases, P53 remains crucial in halting ES progression. Research has shown that ES progression may be linked to the suppression of P53 activity by EWS-FLI1 (31). Ban et al. demonstrated that silencing EWS-FLI1 reactivates NOTCH signaling, which then activates TP53, leading to cell cycle arrest in ES cells (32). Consequently, the TP53 pathway is essential in curbing ES advancement. Stolte et al. identified several genes that TP53 wild-type ES cell lines depend on, including MDM2, MDM4, USP7, and PPM1D (33). Therefore, targeting these genes could be a promising therapeutic approach.

Sonnemann et al. reported that the MDM2 inhibitor Nutlin-3 selectively upregulated P53 gene and protein expression in wild-type P53 Ewing sarcoma cells, leading to significant apoptosis induction. However, Nutlin-3 only inhibited growth in cells with mutant P53 (34). Despite these promising preclinical effects, Nutlin-3 was not advanced to clinical trials due to its limited efficacy and associated toxicity (35). Conversely, ALRN-6924 has emerged as a potent inhibitor of MDM4 with high affinity for MDM2, functioning as a dual MDM4/2 inhibitor. ALRN-6924 activates wild-type P53 during treatment and demonstrates strong antitumor activity in cancer cell lines overexpressing MDM4. Additionally, ALRN-6924 has shown potential in reducing chemotherapy-induced anemia and thrombocytopenia (36). Currently, ALRN-6924 is undergoing phase I clinical trials in patients with solid tumors and lymphomas harboring wild-type TP53, with preliminary results indicating good tolerability and antitumor efficacy (37). Moreover, Schauer et al. identified an irreversible inhibitor, XL177A, targeting USP7. XL177A has demonstrated high sensitivity in ES, effectively binding to USP7 and exhibiting over tenfold selectivity for USP7 compared to closely related deubiquitinating enzymes. XL177A significantly elevates P53 levels, thereby inhibiting cancer cell proliferation. However, mutations in TP53 have been shown to predict reduced sensitivity to XL177A (38). Recent advancements have also highlighted the potential of selective PPM1D inhibition as a promising anticancer strategy, either by delaying tumorigenesis or reducing tumor burden. However, further research is needed to assess the effectiveness of currently available PPM1D inhibitors, underscoring the need for the development of more specific agents (39).

The continuous advancement of next-generation sequencing (NGS) technology has significantly enhanced its clinical utility. Unlike traditional polymerase chain reaction (PCR) amplification methods, NGS technology can sequence the entire human genome within a matter of days, representing a substantial improvement over conventional sequencing methods (40). NGS facilitates the comprehensive genetic profiling of tumors, enabling the identification of precise therapeutic targets and the development of personalized treatment regimens (41).

## 4 Conclusions

In summary, RES is a rare and highly aggressive malignancy with a poor prognosis, predominantly affecting children and adolescents. Current therapeutic regimens primarily involve surgical resection combined with postoperative chemotherapy, but their efficacy remains limited for patients with advanced RES. Recently, neoadjuvant therapy following biopsy has gained acceptance as a viable therapeutic strategy, although its effectiveness requires further validation through clinical trials. As research progresses, targeted therapy has gained increased recognition. The development and application of targeted drugs in cancer treatment warrant further investigation in clinical trials. Additionally, NGS has demonstrated its pivotal role in the diagnosis and treatment of cancer. With the ongoing advancement of NGS technology, it holds the potential to more accurately identify actionable mutant genes, thereby facilitating the selection of targeted therapies and improving prognostic assessment in patients with RES.

## Data Availability

The original contributions presented in the study are included in the article/Supplementary Material, further inquiries can be directed to the corresponding author.
